# Leveraging Distance-Based Effectiveness Indicators for Enhanced Behavioral Pattern Discovery in Complex Problem-Solving Assessment

**DOI:** 10.3390/bs16030383

**Published:** 2026-03-06

**Authors:** Pujue Wang, Jiayi Cheng, Hongyun Liu

**Affiliations:** 1 Beijing Key Laboratory of Learning and Cognition, School of Psychology, Capital Normal University, No. 23 Bai Dui Zi Jia, Beijing 100048, China; 2 Beijing Key Laboratory of Applied Experimental Psychology, National Demonstration Center for Experimental Psychology Education (Beijing Normal University), Faculty of Psychology, Beijing Normal University, No. 19 Xin Jie Kou Wai Street, Beijing 100875, China; 3 Research Center for Capacity Building in Educational Assessment and Evaluation (Beijing Higher Education Innovation Center for Philosophy and Social Sciences), Beijing 100875, China

**Keywords:** process data, problem-solving, distance-based effectiveness, N-gram chi-square feature selection, Dynamic Time Warping, K-medoids clustering

## Abstract

Data-driven approaches have emerged as powerful tools for analyzing process data. This study focuses on two data-driven methods: n-gram chi-square feature selection for extracting key action segments and K-medoids clustering combined with Dynamic Time Warping (DTW) distance for identifying behavioral patterns. To address the limitations that arise when applying these methods to complex tasks where ambiguous raw actions often hinder interpretation, this study introduces distance-based effectiveness indicators to enhance both data-driven methods for analyzing actions in the context of complex problem-solving. The research examines how representing action sequences through state effectiveness ($d_{s}$) and transition effectiveness ($\Delta d_{s\to s^{\prime }}$) indicators outperforms the use of raw actions alone within the complex collaborative problem-solving Balance Beam task. Results consistently demonstrated that effectiveness indicators significantly improved the sensitivity of n-gram feature selection, the performance of clustering, and the interpretability of both n-grams and resulting clusters. Specifically, state effectiveness representations ($d_{s}\to d_{s^{\prime }}$) yielded the best outcomes. These findings advocate for the integration of effectiveness indicators into data-driven process analytics to more effectively capture and explain behavioral patterns of problem-solving.

## 1. Introduction

Interactive simulation tasks closely mirror real-life situations and require examinees to demonstrate complex abilities and skills in performing tasks. Large-scale assessment programs, such as the Programme for International Student Assessment (PISA; OECD, 2014) and the Assessment and Teaching of 21st Century Skills (ATC21S; Griffin & Care, 2014), have pioneered the use of computerized interactive tasks to measure problem-solving abilities. These assessments record rich process data from examinee interactions, thereby extending traditional evaluations of cognitive and behavioral performance (Qiao & Jiao, 2018; S. He & Cui, 2025). Process data are recorded as time-stamped sequences of actions that capture how examinees interact with and advance through interactive tasks. Action sequences, as typical process data, have been widely recognized for their capacity to deepen the understanding of problem-solving competencies (Chang et al., 2017; Chen et al., 2019; Q. He et al., 2019a; Li et al., 2024). Due to the inherent complexity of action sequences, specifically their non-standard and semi-structured formats along with highly variable lengths across participants, there has been a surging demand for methodological innovation within the field of psychometrics in recent years (von Davier et al., 2022). Current analytical frameworks can be broadly classified into two primary categories: psychometric modeling and feature extraction. Psychometric modeling conceptualizes action sequences as a continuous series of item responses and leverages extensions of the item response theory to estimate the latent problem-solving abilities (Liu et al., 2018; Xiao et al., 2021; Wang & Liu, 2024). Feature extraction is typically employed to uncover typical behavioral fragments and overarching problem-solving patterns. This paradigm is further divided into theory-driven and data-driven approaches. Theory-driven methods rely heavily on predefined behavioral indicators and scoring rubrics established by experts (Liu et al., 2018; Yuan et al., 2019), a process constrained by the necessity to develop task-specific rules for each specific task. In contrast, data-driven approaches utilize data mining, natural language processing, and other computational techniques to directly extract latent information from action sequences. These methods offer distinct advantages by minimizing the dependence on manual scoring and significantly enhancing the cross-task transferability of analytical algorithms and pipelines. Given these empirical strengths, data-driven techniques have been extensively adopted for process data analysis in problem-solving assessments (Greiff et al., 2015; Teig et al., 2020; Tan et al., 2025).

The majority of existing data-driven techniques have been developed and validated using problem-solving tasks with relatively simple solution pathways, where the meaning of each action for task completion in these contexts is explicit, unambiguous, and easily evaluated. Consequently, researchers can directly subject raw actions to sequential pattern mining to identify characteristic behaviors (Liao et al., 2019; Q. He et al., 2021; Ulitzsch et al., 2022; Jiang et al., 2022), or employ clustering to explore overarching behavioral patterns (Eichmann et al., 2020; Greiff et al., 2018; Q. He et al., 2019b; Kwon & Zhang, 2025). However, when problem-solving assessments become more complex, particularly when multiple solution paths exist and intertwine, a single action in its raw representation can serve entirely different functions depending on the specific problem context. Consequently, performing feature extraction directly on raw actions fails to capture their actual contribution to task completion, rendering it difficult to delineate meaningful behavioral fragments and distinct patterns.

Recent advancements in psychometric modeling necessitate the evaluation of each action’s value prior to modeling. The sequential response model with polytomous effectiveness indicators (SRM-PEI) proposed by Wang and Liu (2024) introduces distance-based effectiveness indicators capable of assessing the effectiveness of every problem state and action within complex problem-solving tasks. The derivation of these indicators not only incorporates contextual information for each action but also aligns with the fundamental problem-solving principle that states and actions closer to the target are inherently superior. Consequently, these effectiveness indicators hold the potential to resolve the aforementioned dilemma of data-driven feature extraction in complex tasks.

To address this challenge, the present study proposes a methodological integration that repurposes distance-based effectiveness indicators, originally designed for psychometric modeling, as a novel feature representation procedure for data-driven feature extraction. Specifically, this study focuses on two representative algorithms: (1) the n-gram chi-square feature selection model (Q. He & von Davier, 2015, 2016), representing the segment extraction paradigm akin to natural language processing, and (2) K-medoids sequence clustering based on Dynamic Time Warping (DTW) distance (Q. He et al., 2023), representing the distance-based paradigm typical of sequence mining. This research employs a real-world complex problem-solving task and its associated empirical dataset to achieve comprehensive validation. Through two successive empirical studies, we examine whether recoding action sequences with the introduced distance-based effectiveness indicators (Wang & Liu, 2024) enhances the performance of both the n-gram chi-square feature selection and the DTW-based K-medoids sequence clustering in complex problem-solving assessments.

This paper is structured as follows. Section 2 first introduces the two core algorithms examined in this study, followed by a description of the distance-based effectiveness indicators. Subsequently, Section 3 presents a complex collaborative problem-solving task that serves as the empirical example for the investigations. Study 1 and Study 2 then respectively evaluate how integrating distance-based effectiveness indicators with each of the two algorithms enhances analytical outcomes in this task. The paper concludes with Section 6 that synthesizes the main findings and considers their theoretical and practical implications.

## 2. Methods

This section is organized into three parts. Section 2.1 and Section 2.2 introduce the core algorithms of the two analytical pipelines to be enhanced in this study, along with the challenges they face when applied to complex problem-solving tasks. Section 2.3 presents the distance-based effectiveness indicators, which are specifically designed for complex problem-solving tasks and will be utilized to improve the aforementioned analytical approaches.

### 2.1. N-Gram Chi-Square Feature Selection Model

Inspired by practices in natural language processing, sequences of n consecutive actions in process data are termed n-grams. Q. He and von Davier (2015, 2016) employed the chi-square selection model to identify n-grams associated with different task outcomes. First, the TF-ISF (term frequency-inverse sequence frequency) for each n-gram is calculated as:

$$TFI-SF=\{\begin{array}{c} \left[1+\log\left(tf_{i}\right)\right]\times \log\left(\frac{N}{sf_{i}}\right)\;\;\;\;\;if\;tf_{i}\geq 1\\ 0\;\;\;\;\;\;\;\;\;\;\;\;\;\;\;\;\;\;\;\;\;\;\;\;\;\;\;\;\;\;\;\;\;\;\;\;\;\;\;\;\;\;\;\;\;\;\;\;\;\;if\;tf_{i}=0 \end{array}$$

where $tf_{i}$ represents the actual frequency of the *i*-th n-gram, *N* represents the total number of sequences, and $sf_{i}$ represents the number of sequences containing the *i*-th n-gram. This weighting scheme assigns a lower weight to n-grams that are more likely to be produced. For the chi-square feature selection, the rows consist of the *i*-th n-gram versus all other n-grams, and the columns correspond to sequences that completed the task versus those that did not. The chi-square value is calculated for this 2 × 2 contingency table based on the observed and expected TF-ISF values. The n-gram with the highest chi-square value is the one that best distinguishes between groups who completed the task and those who did not. In practice, utilizing unigrams and bigrams (n = 1 and n = 2) is a common convention to ensure adequate occurrence frequencies for each extracted n-gram. Furthermore, the strategic design of the TF-ISF weighting scheme, coupled with the aggregation of all remaining n-gram weights within the chi-square contingency table, renders this analytical approach highly robust against variations in sequence length and inherent data sparsity.

The n-gram chi-square feature selection model achieves optimal performance when specific contiguous actions serve as strong predictors of task success or failure, producing both high chi-square values and readily interpretable features. This condition is typically met in simple tasks where each raw action carries clear meaning and exerts straightforward influence on the outcome, such as the problem-solving tasks in the PIAAC PSTRE domain (see Q. He & von Davier, 2015, 2016 for details). In contrast, complex tasks present substantial challenges, as even minor variations in a single action or subsequence can lead to markedly different effects on task outcomes. In environments characterized by multiple intersecting solution paths, the same raw action may either facilitate or hinder task progress depending on the current intermediate problem state and the subsequent solution path chosen. Without incorporating contextual information, the chi-square model struggles to identify subsequences that critically influence task outcomes. Consequently, researchers face difficulties not only in selecting discriminative features but also in interpreting their meaning and establishing meaningful correlations with other relevant variables.

### 2.2. DTW-Based K-Medoids Clustering

The second method involves transforming the discrepancy information between any two action sequences into a similarity or distance metric. This is followed by distance-based analysis, such as cluster analysis, which aims to cluster participants based on their behavioral patterns. The resulting clusters are typically more aligned with underlying problem-solving success and reveal clearer behavioral typologies.

When calculating sequence similarity for process data analysis, metrics for categorical variable sequences are commonly used, such as edit distance (Hao et al., 2015), the longest common subsequence (Q. He et al., 2019a, 2021), and the dissimilarity measure used by Tang et al. (2020). A more recent method uses Dynamic Time Warping (DTW) to assess the distance between two numerical sequences (Q. He et al., 2023). When two sequences have very similar temporal structures but differ in individual elements or their order, traditional distance measures may fail to capture their similarity. The DTW algorithm finds the optimal warping path between two sequences, calculates the DTW distance, and reflects the similarity between them.

Common techniques applied to the similarity matrix include classical hierarchical clustering (Eichmann et al., 2020; Hao et al., 2015) or K-means clustering (Q. He et al., 2019b). A more recent method employs K-medoids clustering, which is more robust to outliers and often yields more interpretable results (Q. He et al., 2023). This approach allows researchers to examine the problem-solving strategies and behavioral patterns across different participant clusters. Q. He et al. (2023) utilized process data from all students in Rapa Nui reading unit in the PISA 2018 project (OECD, 2021). They calculated the DTW distance for each student’s browsing behavior sequence and time sequence, constructing two sequence similarity matrices. Using K-medoids cluster analysis, they identified four typical navigation patterns and found significant associations between the navigation patterns and students’ reading performance and socio-demographic characteristics.

For the DTW-Based Clustering, challenges also arise. Similarly to the applicable scope of the n-gram chi-square feature selection model, in the PISA 2018 reading Rapa Nui task—a relatively simple one—the raw actions (e.g., page-turning, scrolling) had clear meanings and effects on task completion, making them easy to sequence and interpret for cluster differentiation. However, when the task is more complex and the meaning of actions is uncertain, this analytical pipeline may negatively impact both the clustering effectiveness and the interpretation of the resulting clusters. When a task is so complex that it has multiple completion paths, encoding the actions in a unique sequence according to the task completion order becomes difficult. If one follows the original procedure of Q. He et al. (2023) by numbering problem states or actions based on the task design pages, these representations essentially function as categorical variables. Consequently, this approach is fundamentally unsuitable for the numerical sequence requirements of the DTW algorithm. Furthermore, sequences of categorical variables lack information about the problem context, reducing the informativeness of DTW distance, which may affect the clustering results.

### 2.3. Distance-Based Effectiveness Indicators

Beyond sequential mining, measurement models represent another major category for leveraging action sequences to characterize examinee and item characteristics (Chen, 2020; Fang & Ying, 2020; Zhan & Qiao, 2022; Han et al., 2022; Xiao & Liu, 2024). For complex interactive situations, a participant performs a series of actions that cause transitions between various problem states, ultimately leading to the target state. Within models built upon transition sequences (interchangeable with “action sequences”^1^), an effectiveness parameter is used to evaluate the value of a transition. In recent developments, Wang and Liu (2024) proposed distance-based effectiveness indicators for both problem states and transitions. Specifically, a problem state receives a higher effectiveness rating if it is situated closer to the target state. Consequently, a transition receives a higher effectiveness rating if it leads to a subsequent state that is closer to the target than the preceding state. This underlying principle is conceptually consistent with the assessment of problem-solving ability in Finite State Automata (FSA) frameworks (Funke, 2001; Funke & Buchner, 2007). Following this logic, the state effectiveness indicator ($d_{s}$) denotes the minimum number of remaining transitions required to accomplish the task from a given state. Correspondingly, the transition effectiveness indicator (${∆d}_{s\to s’}$) quantifies the exact extent to which a specific transition alters this minimum required number of transitions. The formal calculation procedure for these distance-based effectiveness indicators is outlined as follows:

First, the distance-based effectiveness for a problem state is defined as the theoretical shortest distance from that state to task completion—that is, the minimum number of transitions required to reach a target state from the current problem state. For a problem state s, the effectiveness $d_{s}$ is equal to the minimum of the shortest distances to all target states. Assume that *k* target states are reachable from state s, denoted as $s_{target}^{\left(1\right)}$, $s_{target}^{\left(2\right)}$ … $s_{target}^{\left(k\right)}$. The distances from state s to these target states are $d_{s}^{\left(1\right)}$, $d_{s}^{\left(2\right)}$ … $d_{s}^{\left(k\right)}$, respectively. The effectiveness indicator for state s is then $d_{s}=min(d_{s}^{\left(1\right)},d_{s}^{\left(2\right)},\ldots ,d_{s}^{\left(k\right)})$. Second, the distance-based effectiveness of a transition is the change of state effectiveness this transition causes. Denote the transition from problem state s to problem state $s^{\prime }$ as $s\to s^{\prime }$. Let the effectiveness of the two problem states be $d_{s}$ and $d_{s^{\prime }}$. The change in the shortest distance to task completion after the transition $s\to s^{\prime }$ occurs is defined as the distance-based effectiveness indicator for the state transition, denoted as ${∆d}_{s\to s’}=d_{s}-d_{s^{\prime }}$. These two indicators can yield a variety of numerical values across multiple problem-solving tasks, leading them to be termed polytomous effectiveness indicators. To validate the latent problem-solving ability estimated by the measurement model, specifically the SRM-PEI, Wang and Liu (2024) computed the following statistics: The mean value of $\Delta d_{s\to s^{\prime }}$ for each action sequence, which reflects the overall efficiency of the problem-solving process. The proportion of actions with different $\Delta d_{s\to s^{\prime }}$ values, which reveals the relative frequency of actions categorized as moving forward ($\Delta d_{s\to s^{\prime }}=1)$, moving backward ($\Delta d_{s\to s^{\prime }}=-1)$, and standing still ($\Delta d_{s\to s^{\prime }}=0)$. Similarly, they calculated the mean value of $d_{s}$ for the corresponding state sequences, which reflects whether the overall problem-solving process tends to move closer to or further from the final target.

### 2.4. Newly Proposed Analytical Pipeline

This study anticipates that these effectiveness indicators can substantially advance the analysis of process data derived from complex problem-solving tasks through the employment of two data-driven approaches: the n-gram chi-square feature selection method and DTW distance-based sequence clustering. Specifically, building upon these methodological frameworks, two investigative studies are proposed:

Study 1: Enhancing Key Action Segment Extraction with Effectiveness Indicators in Complex Tasks. This study will examine how representing action sequences and state sequences via distance-based effectiveness indicators, compared to using raw action sequences, improves both the sensitivity and interpretability of key action segments extracted through the n-gram chi-square feature selection method.

Study 2: Enhancing DTW Distance-Based Sequence Clustering with Effectiveness Indicators in Complex Tasks. This study will explore how representing action sequences with effectiveness indicators, in contrast to raw sequences, improves the performance of K-medoids clustering. Additionally, it will investigate methods for interpreting the resulting clusters of complex problem-solving sequences through the lens of effectiveness indicators.

**Figure 1:**
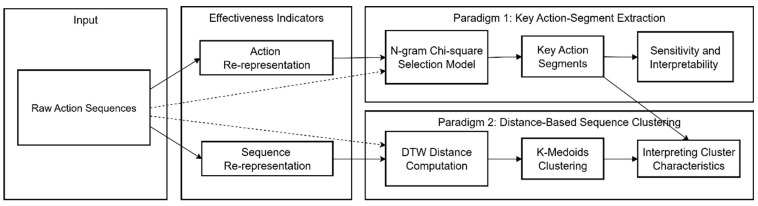
A conceptual framework of the key action-segment extraction paradigm and the distance-based sequence clustering paradigm both enhanced by effectiveness indicators. Dashed lines indicate the original workflow in which raw representations are fed directly into feature extraction, while solid lines denote the proposed workflow, incorporating re-representation based on effectiveness indicators before feature extraction.

Figure 1 presents an integrated pipeline illustrating the key action-segment extraction paradigm and the distance-based sequence clustering paradigm. Both methodological pipelines are systematically enhanced by integrating state and transition effectiveness indicators (i.e., $d_{s}$ and $\Delta d_{s\to s^{\prime }}$) at the action and sequence re-representation levels.

## 3. Empirical Task and Data

**Figure 2:**
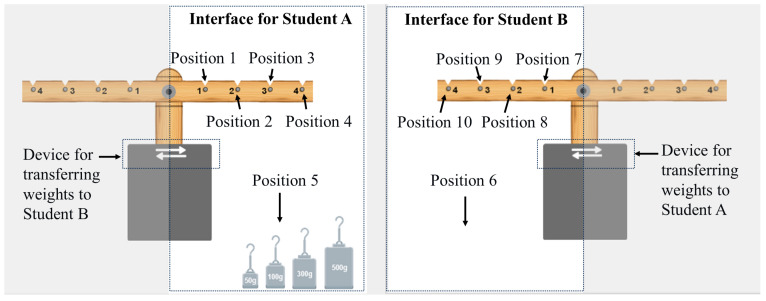
Interface of the Chinese-language version of the balance beam task.

The Balance Beam Task is a collaborative problem-solving task from the Assessment and Teaching of 21st Century Skills project (Griffin & Care, 2014). In this study, we used a Chinese version of the Balance Beam Task developed by Yuan et al. (2019). In this task, two students share four weights (50 g, 100 g, 300 g, and 500 g) and a beam of uniform mass. Each side of the beam features four equally spaced positions for hanging weights, and two students are restricted to hanging weights on their respective sides (see Figure 2). At the initial state of the task, Student A is provided with all four weights, while Student B possesses none, thereby highlighting the inherent role asymmetry between the two participants. Throughout the collaborative process, the students can transfer weights to one another and communicate via a built-in chat interface. To successfully complete the task, the two students must each hang exactly one weight on their respective sides, resulting in a total of exactly two suspended weights. The task is considered successful when the configuration of the weights and their corresponding positions enables the balance beam to achieve a state of equilibrium in accordance with the principle of leverage.

The complexity of this task primarily stems from the multiplicity of target states, as there are various valid combinations of weights and positions that achieve equilibrium. For instance, Student A hanging a 50 g weight at position 2 and Student B hanging a 100 g weight at position 1 constitutes one target state; similarly, Student A hanging a 300 g weight at position 1 and Student B hanging a 100 g weight at position 3 represents another valid target state. Furthermore, this complexity is compounded by the abundance of optimal pathways. Different target states necessitate distinct implementation paths, and even within the pursuit of a single target state, the sequence of actions is frequently interchangeable. For example, to reach the first target state, the actions of Student A passing the 50 g weight to Student B and Student A hanging the 100 g weight at position 1 can be executed in reverse order without impeding task completion. A third dimension of complexity arises from the continuous intersection of these numerous target states and solution pathways, which enables participants to switch strategies dynamically. For example, if Student B moves the 100 g weight from position 1 to position 2, the group effectively shifts their problem-solving trajectory from pursuing the first target state to the second. Moreover, the combinatorial nature of the four weights generates a vast problem space comprising 10,000 distinct problem states and 168,000 possible transitions between them. Consequently, these characteristics make it exceedingly difficult to construct a comprehensive transition diagram encompassing all states or to subjectively evaluate the effectiveness of a raw action.

**Figure 3:**
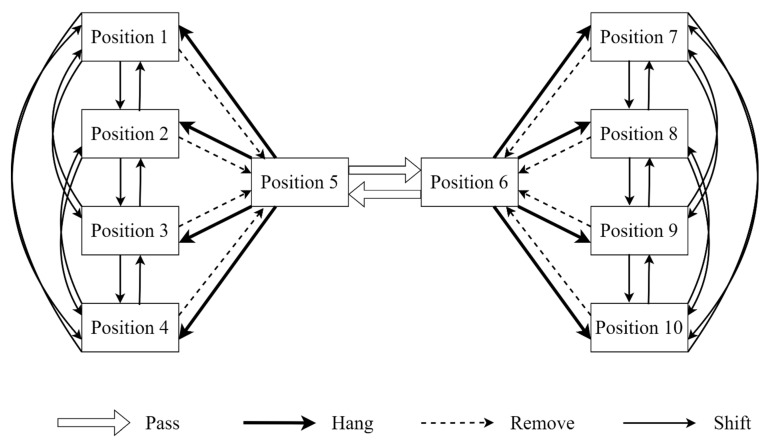
Diagram for four types of actions (transitions) that can occur when a weight moves among ten possible positions in the balance beam task.

The process data were obtained from Yuan et al. (2019), who administered the assessment across eight middle schools located in Beijing, Jiangxi, and Guizhou, China. These specific regions were selected to represent diverse levels of economic development and educational quality. Through cluster random sampling of intact eighth- and ninth-grade classrooms, a total of 422 groups (844 students) were formed. The participants ranged in age from 13 to 15 years (M = 13.91, SD = 0.33), with males comprising 54.5% of the sample. During data preprocessing, the analytical dataset was restricted to four categories of transitions critical to task completion: passing, hanging, removing, and shifting weights. These actions are uniquely capable of inducing valid transitions between problem states (see Figure 3). Aberrant actions stemming from system errors (e.g., weights failing to move as theoretically expected in Figure 3), alongside task-irrelevant actions that failed to elicit any state transitions, were systematically discarded. Ultimately, this rigorous filtering procedure retained 422 continuous and logically valid action sequences. The average sequence length was 49.5, and the overall task completion rate was 39.5%.

Following the procedure outlined by Wang and Liu (2024), the state effectiveness $d_{s}$ and the transition effectiveness $\Delta d_{s\to s^{\prime }}$ were computed for the Balance Beam Task. The effectiveness $d_{s}$ for all intermediate states takes values from 1 to 5, indicating that achieving a balanced state requires a minimum of 1 to 5 steps. The terminal balanced state has a $d_{s}=0$. The effectiveness of each transition is computed using the formula ${∆d}_{s\to s’}=d_{s}-d_{s^{\prime }}$. Transitions between all intermediate states and the balanced state result in three outcomes: a step toward the target ($\Delta d_{s\to s^{\prime }}=1$), no change in distance to the target ($\Delta d_{s\to s^{\prime }}=0$), or a step away from the target ($\Delta d_{s\to s^{\prime }}=-1$). Based on the two effectiveness indicators, each transition can be represented in two ways: one directly uses the effectiveness rating $\Delta d_{s\to s^{\prime }}$ of the transition, and the other represents the transition by the pair of state effectiveness values before and after the action, denoted as $d_{s}\to d_{s^{\prime }}$. This paired coding scheme generates a total of 16 specific representations, which can be further classified into three strategic directions. Specifically, there are five representations of progressive moves where the state distance to the target decreases (i.e., 1→0, 2→1, 3→2, 4→3, and 5→4). Additionally, there are six representations of neutral moves where the state distance remains unchanged (i.e., 0→0, 1→1, 2→2, 3→3, 4→4, and 5→5). Lastly, there are five representations of regressive moves where the state distance increases (i.e., 0→1, 1→2, 2→3, 3→4, and 4→5).

## 4. Study 1: Enhancing Key Action Segment Extraction with Effectiveness Indicators in Complex Tasks

In complex problem-solving tasks, the representation format of the action sequence plays a critical role in the extraction of key actions via the n-gram chi-square feature selection model. Study 1 examines whether representing actions with two types of effectiveness indicators, compared with raw actions, would enhance the sensitivity and interpretability of the n-gram chi-square feature selection model. First, the sensitivity of n-gram chi-square feature selection refers to both the number of key action segments extracted and its discriminative power to distinguish between successful and failure problem-solving groups. Second, the interpretability of key action segments means the extent to which the extracted segments can be easily and meaningfully linked to problem-solving processes.

### 4.1. Research Procedure and Data Analysis in Study 1

Considering computational complexity and actual frequency of occurrence, this study focuses on both unigrams (1-g, single actions) and bigrams (2-g, two consecutive actions). Each action in an n-gram was represented in three representations: (1) The raw action is directly extracted from the process data. (2) Each action is represented by the numerical value of its corresponding distance-based transition effectiveness $\Delta d_{s\to s^{\prime }}$. (3) Each action is represented by the effectiveness $d_{s}$ of the state before and after the transition (i.e., $d_{s}\to d_{s^{\prime }}$), forming a pair such as 3 → 2. Table 1 presents one example each of 1-g and 2-g under the three representations.

Regarding sensitivity, beyond the mere quantity of generated n-grams, each n-gram is statistically evaluated by computing its chi-square (χ^2^) value and phi (φ) coefficient derived from a 2 × 2 contingency table. Higher values for both metrics indicate a stronger capacity of the given n-gram to distinguish between successful and failure groups. Concurrently, these chi-square values undergo significance testing (*df* = 1, *α* = 0.05). To mitigate the potential false discovery rate, the Benjamini–Hochberg procedure is applied across all n-grams within a single representation type. The n-grams that remain statistically significant following this correction are designated as key action segments. To comprehensively compare the three representations, the absolute number and the proportion of significant n-grams yielded by each representation were calculated. Furthermore, the average chi-square values and mean phi coefficients across all extracted n-grams are compared among the three representations.

With respect to interpretability, two criteria were examined sequentially for each representation. First, from the perspective of the task context and problem-solving principles, it was evaluated whether each n-gram could be assigned a clear and consistent objective interpretation regarding its function and value. If such an objective evaluation was not attainable, the extent to which consistent subjective judgments could be reached through human evaluation was assessed. Second, once either objective or subjective evaluations were established, the consistency between the binary classification of an n-gram as beneficial or detrimental and its empirical frequency pattern in the chi-square selection model was examined, specifically whether it occurred more frequently in the successful groups (as indicated by the plus sign following each n-gram in Table 2).

### 4.2. Study 1 Results

The results are presented sequentially, first for 1-g and then for 2-g, followed by a systematic comparison of the sensitivity and interpretability across the three representations. Since only one student initially possesses all the weights, the roles two students play and the strategies they employ lead to different effects on the problem-solving process for the same action; therefore, it is necessary to distinguish roles for the n-gram chi-square feature selection.

With respect to the sensitivity of 1-g selection, the state effectiveness representation ($d_{s}\to d_{s^{\prime }}$) exhibited the highest sensitivity. Although its total number of extracted 1-g is lower than that of the raw representation, it achieves the highest performance across all other metrics. Specifically, the absolute number of key segments, the proportion of key segments to the total extracted segments (81.3%), and the average chi-square values and phi coefficients for both total and key segments are the highest among the three representations (see Table 2). The raw representation ranked second in overall sensitivity. This method successfully generates the highest total number of distinct 1-g. However, the absolute number of key segments, the proportional yield of key segments (9.7%), and the average chi-square values and phi coefficients all rank second in the comparative analysis. Finally, the transition effectiveness representation ($\Delta d_{s\to s^{\prime }}$) demonstrated the lowest sensitivity. It yields the fewest 1-g and produces the lowest average chi-square values and phi coefficients. Notably, none of the segments extracted under this specific representation passed the statistical significance test.

Regarding the interpretability of 1-g across the three representations, numerous actions within the raw representation necessitate a predefined problem state to accurately determine their utility for task completion. The evaluative outcomes for certain actions dynamically shift according to structural changes in the problem state, thereby rendering a unified, deterministic, and objective assessment highly challenging. Using the 1-g with the highest chi-square values as illustrative examples (see Table 3), the first two actions involve Student B hanging a 500 g weight at position 2 or position 3. Because this task has no balanced state that incorporates a suspended 500 g weight, both actions are demonstrably detrimental to task completion. Conversely, an action such as “Student B removes the 100 g weight from position 1” occurs more frequently within the successful groups. However, when situated within the specific problem state previously illustrated in Table 1, this action proves highly detrimental to task completion. Furthermore, this detrimental nature is exclusively revealed with the help of effectiveness indicators. Prior to this action, only two steps remained to complete the task; following the action, the required number of steps increased to three. However, if the problem state is altered to a hypothetical scenario where Student A has hung a 300 g weight at position 1 and Student B has hung a 100 g weight at position 1, the utility of this identical action is no longer negative. To obtain a definitive evaluation for each 1-g within the raw action representation, reliance on subjective assessment becomes mandatory. Three domain experts, highly familiar with the task mechanics, evaluated the utility of the 72 extracted 1-g on a continuous scale from 1 to 5. The final Kendall coefficient of concordance (Kendall’s W) among the raters reached 0.785.

In contrast, all 1-g derived from the two effectiveness indicator representations inherently possess definitive and objective evaluations of their utility. Consequently, their objective consistency is considered to be 100%. The numerical values and corresponding mathematical signs of these effectiveness metrics explicitly indicate how each 1-g alters the minimum number of transitions required for task completion, completely eliminating the need for manual human evaluation. In summary, regarding the consistency of direct subjective or objective evaluation for interpretability, the state effectiveness representation ($d_{s}\to d_{s^{\prime }}$) was comparable to the transition effectiveness representation ($\Delta d_{s\to s^{\prime }}$), and both are significantly superior to the raw action representation.

Regarding the second criterion, the majority consensus for the raw representation among three experts was established with the midpoint score of 3 as the definitive cutoff threshold. When this subjective expert consensus was compared against the chi-square selection model’s indication of higher frequency for the successful groups, the overall consistency rate was a mere 27.3%. For two effectiveness representations, a specific challenge arises because both effectiveness indicators can generate a value of zero, signifying no change in the shortest path required to complete the task. If this zero value is classified as detrimental to task completion and assigned a negative sign, the consistency with the sign of the chi-square selection model reaches 100% for the transition effectiveness representation ($\Delta d_{s\to s^{\prime }}$), whereas the consistency for the state effectiveness representation ($d_{s}\to d_{s^{\prime }}$) stands at 57.1%. In summary, regarding the consistency with the relative frequency between the success and failure groups, the transition effectiveness representation ($\Delta d_{s\to s^{\prime }}$) outperforms the state effectiveness representation ($d_{s}\to d_{s^{\prime }}$), which in turn outperforms the raw action representation.

A detailed analysis of the 1-g from these two effectiveness representations reveals the specific behavioral patterns associated with completing this task. In terms of $\Delta d_{s\to s^{\prime }}$, the successful groups exhibited more target-approaching actions ($\Delta d_{s\to s^{\prime }}>1$) and fewer instances of “standing still” ($\Delta d_{s\to s^{\prime }}=0$) or moving away from the target ($\Delta d_{s\to s^{\prime }}<1$). For the $d_{s}\to d_{s^{\prime }}$ representation, moderate consistency does not imply limited information. As shown in Table 3, the three transitions with the highest chi-square values demonstrate clear and consistent patterns: target-approaching actions occurred more frequently in the successful groups (e.g., 1→0 for both Students A and B), whereas “standing still” transitions were more prevalent in the failure groups (e.g., 4→4 for Student A). Because the initial state of this specific task requires a minimum of three steps for completion, these stagnant transitions fundamentally constitute erroneous attempts. Furthermore, statistical inconsistencies also stem from trial-and-error behaviors, such as 1→1 transitions for both students, which occur at the penultimate stage of the task. Although such actions involve temporary stagnation, chi-square selection indicates that they appear more frequently in the successful groups. This pattern reflects a fundamental characteristic of the task: successful groups identify the correct path toward the target and can afford exploratory attempts along the way. Conversely, the failure groups fail to identify the correct path and frequently deviate further from the target.

During the extraction of 2-g, the state effectiveness format ($d_{s}\to d_{s^{\prime }}$) remains the most sensitive representation. It generates the second highest total number of 2-g. Furthermore, it achieves the highest absolute number and proportion of significant key segments (25.6%), alongside the highest average chi-square values and phi coefficients for both the total extracted segments and the key segments. The transition effectiveness representation ($\Delta d_{s\to s^{\prime }}$) yields the lowest total number of 2-g, a substantial 22.2% of these extracted bigrams constitute significant key segments. Its average chi-square values and phi coefficients closely approximate those of the state effectiveness representation, thereby establishing it as the second most sensitive format. Conversely, the raw action representation extracts the highest total number of segments. However, this specific method produces the lowest average chi-square values and phi coefficients, and it completely fails to generate any significant key segments.

From the interpretability, key 2-g represented by raw actions remained difficult to interpret due to the lack of information about the positions of other weights. Their meaning aligned with their relative frequency in the successful groups only under the assumption that no other weights were placed. For instance, the key 2-g exhibiting the highest chi-square value, specifically “Student A passes the 100 g weight to Student B; Student B hangs the 100 g weight at position 3,” reliably leads to a balanced state and a correspondingly higher frequency in the successful groups as indicated by the chi-square selection model only if the 50 g and 500 g weights are absent from the beam. Conversely, under the specific problem state previously illustrated in Table 1, this exact bigram is demonstrably detrimental to task completion. Notably, formulating this negative judgment still inherently requires the calculation results from the effectiveness indicators. Overall, not every 2-g possesses a uniquely deterministic evaluation outcome. Consequently, the Kendall’s coefficient of concordance among the subjective evaluations provided by the three domain experts reached only 0.535. In stark contrast, the two effectiveness representations successfully incorporate contextual information from target-approaching perspectives, thereby achieving a 100% consistent objective evaluation result and significantly superior to the raw action representation.

For the second criterion, when utilizing the majority consensus from the subjective expert evaluations for the raw action representation, the consistency with the chi-square selection model drops to a mere 13.7%. For both effectiveness-based representations, the effectiveness value for a 2-g was computed as the average of the effectiveness values of the two constituent transitions. The consistency between this average effectiveness and the chi-square model regarding higher frequency within the successful groups reaches 72.2% for the $d_{s}\to d_{s^{\prime }}$ representation and 54.3% for the ${\Delta d}_{s\to s'}$ representation which subsequently outperforms the raw action representation.

The key 2-g segments derived from the two effectiveness-based representations continued to capture salient behavioral characteristics of the task. The highest relative-frequency 2-g in the successful groups predominantly feature both participants making collaborative forward progress (e.g., “A: 2→1; B: 1→0”, as well as “A: 1; B: 1” and “B: 1; A: 1”). In contrast, the most frequent 2-g in the failure groups typically indicated repeated trial-and-error by a single role, such as persistent stagnation (e.g., “A: 4→4; A: 4→4” and “B: 3→3; B: 3→3”).

## 5. Study 2: Enhancing DTW Distance-Based Sequence Clustering with Effectiveness Indicators in Complex Tasks

Study 2 focuses on the application of K-medoids clustering with Dynamic Time Warping (DTW) distance to classify behavioral patterns in complex problem-solving tasks, expanding upon recent methodological advances (Q. He et al., 2023). This study addresses two primary research objectives. The first objective was to investigate whether sequence representations with two types of distance-based effectiveness indicators, paired with DTW distance, yield more behaviorally meaningful and diagnostically informative clusters compared to sequences encoded with raw actions, which lack contextual information and consist of non-numerical variables. The second objective was to develop a more interpretable pipeline for explaining the resulting clusters. In previous process-based studies, the interpretation of clustering relied on comparing the frequencies of raw actions across clusters. Study 2 also explores whether action frequencies under effectiveness indicator representations can better explain clustering results than raw actions.

### 5.1. Research Procedure and Data Analysis in Study 2

The Balance Beam task and empirical data used for comparison in Study 2 are identical to those in Study 1. The difference is that the unit of analysis in Study 2 is the complete sequences without role differentiation to preserve the properties of the numerical sequences. The sequences formed by the three representations are as follows:

(1) Raw sequence: To closely follow the procedure of Q. He et al. (2023), this study utilized the positional coding and transition graph for problem states in the Balance Beam task summarized by Wang and Liu (2024). Based on all actions illustrated in Figure 3, a total of 84 possible raw actions across the two roles were sequentially coded from 1 to 84.
(2) State effectiveness ($d_{s}$): A sequence was formed using the $d_{s}$ value of the problem state after each transition. For example, the optimal problem-solving process produces the sequence “2, 1, 0”. Since the initial state of this task has a $d_{initial}=3$, meaning the task can be solved in a minimum of 3 steps.
(3) Transition effectiveness ($\Delta d_{s\to s^{\prime }}$): A sequence was formed using the numerical value of the effectiveness $\Delta d_{s\to s^{\prime }}$ for each transition. For example, the optimal problem-solving process is encoded as “1, 1, 1”.

Following the re-encoding of the sequences, the analytical procedure proceeds in three distinct steps: First, for the 422 sequences under the three encodings, distance matrices (422 × 422) were computed using the Dynamic Time Warping (DTW) algorithm. Prior to computation, the raw action sequences required conversion into numerical sequences for DTW calculation, whereas the two types of sequences represented by effectiveness indicators could be directly computed as numerical sequences. Dynamic time warping distances were computed using the dist() function within the dtw (version 1.23-1) R package.

Second, K-medoids clustering was performed based on the distance matrices. This procedure was executed via the pam() function from the cluster (version 2.1.8) R package with default settings.^2^ The silhouette index and the Calinski–Harabasz index serve as key metrics for evaluating clustering quality and determining the optimal number of clusters. Ranging from −1 to +1, the silhouette index quantifies how well each data point aligns with its assigned cluster compared to other clusters, with higher values indicating better-defined clusters (Rousseeuw, 1987). Calinski–Harabasz (CH) index calculates the ratio of between-cluster dispersion to within-cluster dispersion. Accordingly, larger numerical values strictly indicate more cohesive and well-separated cluster configurations (Caliński & Harabasz, 1974).

Third, to systematically interpret the behavioral characteristics of the identified clusters, several external criteria were incorporated into the analysis. Specifically, these criteria encompass the 1-g extracted under the three representations in Study 1, the final task performance, the action sequence length, the average sequence-level state and transition effectiveness, and the latent problem-solving ability estimated by the SRM-PEI psychometric model.

### 5.2. Study 2 Results

**Figure 4:**
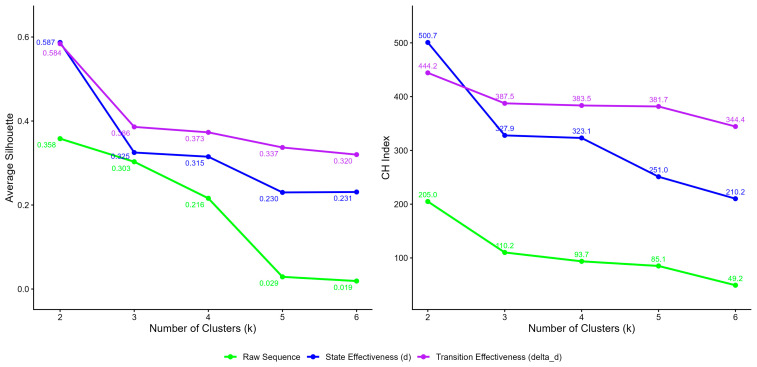
The silhouette index and Calinski–Harabasz index for DTW-based K-medoids clustering across different numbers of clusters under the three sequence representations.

As the number of clusters increased from two to six, the silhouette and CH indices for all three sequence types consistently declined (see Figure 4), and therefore larger numbers of clusters were not attempted. As a result, the two effectiveness-indicator sequences demonstrated superior clustering performance relative to the raw action sequences, particularly with two clusters. The optimal solution was the two-cluster solution that achieved the highest silhouette index (0.587) and CH index (500.7), which was obtained by clustering the state effectiveness ($d_{s}$) sequences into two clusters. In this result, Cluster 1 contained sequences from 280 groups, while Cluster 2 contained sequences from 142 groups.

To interpret the characteristics and differences between the two clusters, independent samples t-tests (*df* = 420, α = 0.05) were initially conducted to examine the frequency differences in key 1-g across the three representations. Furthermore, the Benjamini–Hochberg procedure was applied to control the false discovery rate. Because the sequence clustering algorithm does not differentiate between participant roles, identical actions executed by both roles were combined during Study 2. Consequently, significant differences were observed for 5 out of the 7 key unigrams under the raw action representation, demonstrating Cohen’s *d* effect sizes ranging from 0.397 to 0.481. Again, due to the ambiguous meaning and effect of raw actions, no consistent trend could be easily found between the two clusters.

**Figure 5:**
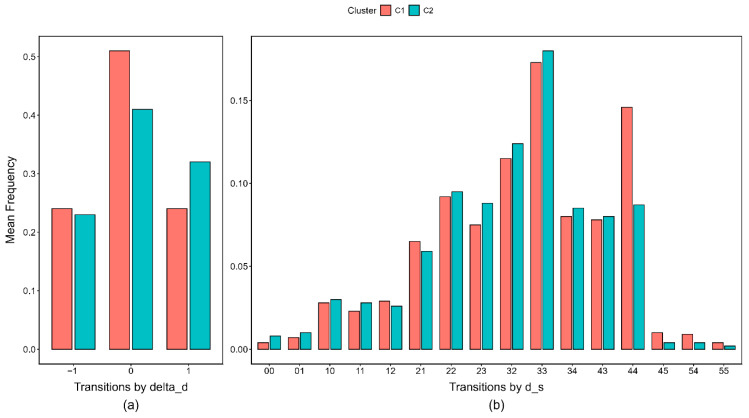
Bar charts of unigram frequency differences between the two clusters across the two effectiveness-based representations. (**a**) Frequency differences for three types of transitions represented by $\Delta d_{s\to s^{\prime }}$; (**b**) Frequency differences for 16 types of transitions represented by $d_{s}\to d_{s^{\prime }}$. The arrow in $d_{s}\to d_{s^{\prime }}$ is omitted on the *x*-axis.

All three types of transitions encoded by $\Delta d_{s\to s^{\prime }}$ yielded statistically significant differences between the two clusters. Specifically, Cluster 1 executed a significantly higher frequency of transitions categorized as “moving away from the target” ($\Delta d_{s\to s^{\prime }}=-1$, Cohen’s *d* = 0.166) and “standing still” ($\Delta d_{s\to s^{\prime }}=0$, Cohen’s *d* = 1.033). Both behavioral patterns are strongly indicative of trial-and-error strategies, thereby reflecting a comparatively lower problem-solving ability coupled with higher task persistence. Conversely, Cluster 2 exhibited a significantly higher frequency of “moving toward the target” transitions ($\Delta d_{s\to s^{\prime }}=1$, Cohen’s *d* = 0.816), strongly suggesting a fundamentally higher problem-solving ability (see Figure 5a).

Regarding the 16 specific transition types encoded by $d_{s}$, statistically significant frequency differences were detected in 14 distinct categories, demonstrating Cohen’s *d* effect sizes ranging from 0.616 to 1.277. Cluster 1 engaged in transitions that led to intermediate states with state effectiveness values exceeding the initial state ($d_{initial}=3$), such as 4→4, 4→5, 5→4, and 5→5 (see Figure 5b). These transitions typically occurred after weights had been placed at positions that could no longer yield balance, and therefore reflected exploration along incorrect paths. This behavioral pattern is highly consistent with a profile of lower ability accompanied by stronger persistence. In contrast, Cluster 2 more frequently engaged in intermediate states with state effectiveness closer to the target state ($d_{target}=0$), such as 1→0 and 1→1, as well as the initial state, such as 3→2, 3→3, and 3→4. These behaviors were aligned with the beginning of the correct solution pathway or with exploration at the final step before completion, further supporting the interpretation of higher ability.

The remaining external validity indicators comprehensively corroborated these distinctive characteristics for both identified clusters. Compared to Cluster 2, Cluster 1 demonstrated higher persistence, evidenced by significantly longer sequence lengths (*p* < 0.001, Cohen’s *d* = 2.667). However, Cluster 1 exhibited a significantly lower task success rate (*p* = 0.035, Cohen’s *d* = 0.094). Across both effectiveness-based metrics, Cluster 1’s sequences were, on average, further from the target state, as indicated by a lower mean $d_{s}$ (*p* < 0.001, Cohen’s *d* = 0.571) and a lower mean $\Delta d_{s\to s^{\prime }}$ (*p* < 0.001, Cohen’s *d* = 0.571). Consistently, SRM-PEI estimates suggested that Cluster 1 also had lower latent problem-solving ability (*p* < 0.001, Cohen’s *d* = 0.369).

## 6. Discussion

### 6.1. Conclusions and Discussion

The current research systematically enhances two distinct data-driven feature extraction methodologies, specifically the n-gram chi-square selection model and Dynamic Time Warping (DTW) distance-based K-medoids sequence clustering, for the rigorous analysis of action sequences generated during complex problem-solving tasks. Each method was validated through a corresponding empirical study, and the results consistently demonstrated the advantages of using the two distance-based effectiveness indicators over using raw actions alone in feature extraction and clustering. Specifically, representations by state effectiveness $d_{s}$ and transition effectiveness $\Delta d_{s\to s^{\prime }}$ enhanced the sensitivity of extracting key actions, the ability to differentiate behavioral patterns across groups, and the interpretability of both two data-driven methods. These improvements stem from the value assessments provided by effectiveness indicators, which enrich contextual information available for each action. Synthesizing the results across the two data-driven approaches, the research identified the recommended sequence representations and interpretive strategies for analyzing action logs generated in complex problem-solving tasks.

Study 1 suggested that using distance-based effectiveness representations, especially the $d_{s}\to d_{s^{\prime }}$ format, provided the optimal trade-off between sensitivity and interpretability for extracting key behavioral segments. This representation produced the largest number of key segments and the highest chi-square value and phi coefficient. This is primarily attributed to the fundamental mechanism of effectiveness indicators, which not only assign explicit qualitative evaluations to actions but also systematically categorize these actions based on their specific numerical values. Consequently, the state effectiveness representation $d_{s}\to d_{s^{\prime }}$ achieves a notably high occurrence frequency while simultaneously demonstrating substantial frequency discrepancies between successful and failure groups. Furthermore, the transition effectiveness representation ($\Delta d_{s\to s^{\prime }}$) exhibits an exceptionally strong categorization capacity, yielding a constrained set of permissible values and fewer distinct categories. Therefore, it demonstrates slightly weaker statistical sensitivity, yet it remains highly advantageous regarding explicit interpretability. Conversely, the raw action representation proved strictly the weakest in both statistical sensitivity and analytical interpretability. This methodological deficiency is particularly pronounced for 2-g, primarily because the excessive variety of action types intrinsically leads to severely low occurrence frequencies. Additionally, the ambiguous and non-unique utility of raw actions ultimately fails to manifest any significant behavioral differences between successful and failure problem-solving groups.

Study 2 showed that distance-based effectiveness representations not only improved the performance of distance-based clustering but also served as meaningful behavioral indicators for interpreting the clustering results. Sequences represented by the two types of effectiveness indicators, yielded higher silhouette and CH indices, indicating that sequences were more closely aligned with their assigned clusters and more distinct from other clusters. Regarding interpretability, two distance-based effectiveness representations $d_{s}\to d_{s^{\prime }}$ and $\Delta d_{s\to s^{\prime }}$ were particularly suitable for interpreting behavioral differences between clusters. Their advantages included both an optimal quantity of transition types and the explicit interpretability of these transitions. Consequently, this methodological combination significantly facilitated the interpretation of cluster-specific behavioral patterns and comprehensively aligned with the cluster characteristics revealed by external validity indicators. By contrast, raw actions proved significantly less effective for yielding optimal clustering configurations and functioning as behavioral indicators to interpret cluster characteristics.

This research systematically investigated the impact of sequence representations on different types of data-driven methods. The results from the two studies highlight five advantages of using effectiveness indicators over raw actions in complex problem-solving tasks: (1) direct improvements in algorithmic performance, as evidenced by higher chi-square values for key n-grams in Study 1 and higher clustering validity in Study 2; (2) clearer and more interpretable features, reflected in the improved interpretability of key actions in Study 1 and the ability to summarize meaningful behavioral patterns in Study 2; (3) greater sensitivity to behavioral differences across groups, demonstrated both by the higher chi-square values of key n-grams in Study 1 and their significant frequency differences across clusters in Study 2; (4) stronger associations between extracted features and external variables, indicated by the use of key n-grams extracted in Study 1 to interpret the clustering results obtained in Study 2; and (5) automated rule-based programming executes distance-based effectiveness indicators within milliseconds. Meanwhile, by substantially reducing the total volume of generated n-grams, the comprehensive feature extraction process accelerates to require merely minutes. In contrast, using raw actions is extremely time-consuming when computing chi-square selection for 2-g. Collectively, these five advantages derive from the fact that effectiveness indicators provide a meaningful numeric value for each state or action, adding the contextual information and linking it to the problem-solving ability, thereby improving the data-driven feature extraction.

### 6.2. Limitations and Future Directions

Several limitations of this study should be acknowledged, particularly regarding the generalizability of the findings. First, the demonstrated advantages of effectiveness-enhanced representations over raw action representations may be inherently tied to the specific task design, characterized by multiple intersecting problem-solving paths. In this type of task, the same raw action can occur within different problem-solving paths and have different effects depending on the intermediate states. This inherent ambiguity of raw actions motivated the introduction of effectiveness indicators to enrich the contextual information. In contrast, if a specific task has a single optimal solution pathway, or if actions across multiple pathways remain entirely distinct, thereby enabling raw actions to possess objective, consistent, and easily evaluable utility (e.g., the PISA 2012 Tickets task; OECD, 2014), the existing data-driven methods and analytical pipelines based on raw action sequences may inherently suffice. Furthermore, the theoretical reliance on shortest-path optimality for effectiveness indicators presents an inherent limitation when evaluating diverse cognitive strategies in different problem-solving contexts, since trial-and-error actions are not merely frequent but often essential and inevitable. This characteristic is particularly pronounced in alternative assessment frameworks, such as linear structural equation (LSE) tasks, which explicitly require repeated exploration to ascertain the complex relationships between input and output variables (for a recent application of effectiveness coding in the LSE task, see Han et al., 2025). Consequently, whether the integration of effectiveness indicators can further enhance analytical performance under the various conditions mentioned above requires additional empirical verification. Second, this study did not account for erroneous termination conditions, and actions irrelevant to the core problem-solving process were excluded from the primary analysis. For these actions, the computation of distance-based effectiveness indicators might present methodological challenges, ultimately necessitating alternative effectiveness indicators or more sophisticated data processing procedures. In summary, the extent to which the current findings generalize across diverse tasks, data-driven methodologies, and strategies for incorporating effectiveness indicators remains a critical open question for future research. Third, regarding the DTW computation, the current analytical pipeline relied exclusively on the default algorithmic configurations. Specifically, the potential impacts of alternative parameter settings, such as sequence normalization procedures and specific warping constraints, on the final distance calculations were not systematically explored. Another important consideration is that computing the distance matrix for all sequences is computationally intensive, which may limit the applicability of distance-based sequence clustering to large-scale assessment datasets. Employing more efficient distance computation algorithms, or alternatively, clustering effectiveness-encoded sequences directly without distance calculation, represents a promising direction for future research.

This study is the first to apply effectiveness indicators, which originated from process-based measurement modeling, to data-driven feature extraction. While this research integrates effectiveness indicators specifically during encoding and interpretation, future studies should explore other methodological phases, particularly optimizing computationally expensive large-N n-gram extractions and sequence distance matrix calculations. Furthermore, the application of state and transition effectiveness indicators extends beyond these specific methods. They serve as foundational components improving diverse models in process data analysis (Goldhammer et al., 2021). Finally, computational psychometrics strongly advocates combining such indicators with hierarchical analytic approaches, seamlessly integrating feature extraction, representation, and psychometric modeling (von Davier et al., 2022).

## Figures and Tables

**Table 1:** Examples of 1-g and 2-g across three representations.

Representation	1-g	2-g
		Action 1; Action 2
Previous State	Student B has a 100 g weight hung at position 1 and a 300 g weight hung at position 2, while Student A holds the 50 g and 500 g weights unhung.	Student A has a 500 g weight hung at position 2 while holding the 50 g and 100 g weights, whereas Student B holds the remaining 300 g weight.
Raw action	Student B removes the 100 g weight from position 1.	Student A passes the 100 g weight to Student B; Student B hangs the 100 g weight at position 3.
$\Delta d_{s\to s^{\prime }}$	B: −1	B:0; A:0
$d_{s}\to d_{s^{\prime }}$	B:2→3	B:3→3; A:3→3

**Table 2:** Counts of total and key segments, mean chi-square values, and mean phi coefficients across three representations for 1-g and 2-g.

Representation	Total Extracted Segments			Significant Key Segments		
	Count	χ^2^	φ	Count	χ^2^	φ
1-g						
Raw action	72	2.817	0.063	7	13.697	0.174
$\Delta d_{s\to s^{\prime }}$	32	34.824	0.247	26	42.362	0.289
$d_{s}\to d_{s^{\prime }}$	6	1.555	0.045	0	-	-
2-g						
Raw action	2999	0.126	0.012	0	-	-
$\Delta d_{s\to s^{\prime }}$	199	5.400	0.080	51	17.611	0.193
$d_{s}\to d_{s^{\prime }}$	36	4.909	0.080	8	16.400	0.188

**Table 3:** Top three 1-g and 2-g with the highest chi-square values across three representations.

Representation	N-Gram	χ^2^	φ
1-g			
Raw Action	Student B hangs the 500 g weight at position 2	34.039	0.284
	Student B hangs the 500 g weight at position 3	11.975	0.168
	Student B removes the 100 g weight from position 1 (+)	11.684	0.166
$d_{s}\to d_{s^{\prime }}$	A: 4→4	150.799	0.598
	B: 1→0 (+)	121.979	0.538
	A: 1→0 (+)	97.091	0.480
$\Delta d_{s\to s^{\prime }}$	B: 1 (+)	6.556	0.125
	A: 0	1.831	0.066
	B: 0	0.479	0.034
2-g			
Raw Action	Student A passes the 100 g weight to B; Student B hangs the 100 g weight at position 3 (+)	5.580	0.115
	Student A hangs the 300 g weight at position 4; Student A hangs the 100 g weight at position 3	5.413	0.113
	Student A hangs the 300 g weight at position 1; Student B hangs the 100 g weight at position 3 (+)	4.848	0.107
$d_{s}\to d_{s^{\prime }}$	A: 4→4; A: 4→4	88.129	0.457
	A: 2→1; B: 1→0 (+)	50.380	0.346
	B: 3→3; B: 3→3	46.439	0.332
$\Delta d_{s\to s^{\prime }}$	A:1; B:1 (+)	41.366	0.313
	B:1; A:1 (+)	25.365	0.245
	A:1; A:1 (+)	16.988	0.201

Note: The suffix (+) indicates a higher frequency in the successful groups, whereas segments without this suffix indicate a higher frequency in the failure groups.

## Data Availability

The data presented in this study are available in Open Science Framework (OSF) at https://osf.io/bde69/ (accessed on 23 February 2026).

## Notes

1 In this study, the term “action” is used to denote the behavioral representations recorded in raw log data (e.g., clicking a “next page” button), whereas “transition” describes the subsequent change in the task context induced by that action (e.g., navigating from the first page to the second). Throughout the problem-solving process, problem states remain relatively static, and a “transition” essentially connects two successive problem states. Strictly speaking, not all actions precipitate a transfer in the problem state (e.g., clicking on a blank area of the interface). Nevertheless, such redundant actions typically fall outside the scope of modeling research and are systematically filtered out during data preprocessing. Consequently, within the literature on process data-based measurement models, the terms “action” and “transition” are frequently used interchangeably (e.g., Fu et al., 2024; Wang & Liu, 2024).

2 The datasets and R scripts are publicly accessible via https://osf.io/bde69/.
